# Phenotypic and Genotypic Characterization of Newly Isolated *Xanthomonas euvesicatoria*-Specific Bacteriophages and Evaluation of Their Biocontrol Potential

**DOI:** 10.3390/plants12040947

**Published:** 2023-02-19

**Authors:** Yoana Kizheva, Zoltan Urshev, Melani Dimitrova, Nevena Bogatzevska, Penka Moncheva, Petya Hristova

**Affiliations:** 1 Department of General and Industrial Microbiology, Faculty of Biology, Sofia University St. “Kliment Ohridski”, 8 Dragan Tsankov Blvd., 1000 Sofia, Bulgaria; 2 R&D Department, LB Bulgaricum PLC, 14 Malashevska Str., 1000 Sofia, Bulgaria; 3 Institute of Soil Science, Agrotechnologies and Plant Protection “N. Poushkarov”, Agricultural Academy, 1331 Sofia, Bulgaria

**Keywords:** *Myoviridae* bacteriophages, bacterial spot disease, *Xanthomonas euvesicatoria*, circular genome permutation, rhizosphere

## Abstract

Bacteriophages have greatly engaged the attention of scientists worldwide due to the continuously increasing resistance of phytopathogenic bacteria to commercially used chemical pesticides. However, the knowledge regarding phages is still very insufficient and must be continuously expanded. This paper presents the results of the isolation, characterization, and evaluation of the potential of 11 phage isolates as natural predators of a severe phytopathogenic bacterium—*Xanthomonas euvesicatoria*. Phages were isolated from the rhizosphere of tomato plants with symptoms of bacterial spot. The plaque morphology of all isolates was determined on a *X. euvesicatoria* lawn via a plaque assay. Three of the isolates were attributed to the family *Myoviridae* based on TEM micrographs. All phages showed good long-term viability when stored at 4 °C and −20 °C. Three of the phage isolates possessed high stability at very low pH values. Fifty-five-day persistence in a soil sample without the presence of the specific host and a lack of lytic activity on beneficial rhizosphere bacteria were found for the phage isolate BsXeu269p/3. The complete genome of the same isolate was sequenced and analyzed, and, for the first time in this paper, we report a circular representation of a linear but circularly permuted phage genome among known *X. euvesicatoria* phage genomes.

## 1. Introduction

Bacterial spot in tomato and pepper (caused by *Xanthomonas euvesicatoria* pv. euvesicatoria (formerly known as *Xanthomonas euvesicatoria*), *X. euvesicatoria* pv. perforans (formerly known as *Xanthomonas perforans*), *Xanthomonas hortorum* pv. gardneri (formerly known as *Xanthomonas gardneri*), and *Xanthomonas vesicatoria*) has cyclic peaks. It is an economically important disease and a severely limiting factor for fruit yield in these crops worldwide [1,2,3].

To date, three species (*X. vesicatoria, X. euvesicatoria*, and *X. gardneri*) that infect tomato and two species (*X. vesicatoria* and *X. euvesicatoria*) that infect pepper have been described in Bulgaria. The natural population of pathogens is heterogeneous by symptoms, species, phenotypic and genotypic characteristics, pathotype, and races [4,5,6,7,8,9]. 

The control of the disease and the battle against bacterial spot agents are extremely difficult due to several factors, such as the dynamic changes in the species and race composition; the cyclicity of their natural populations; their symptomatic and symptomless development in hosts and non-hosts; their relationships with the normal microbiota of host plants; their ability to be stored in a hypobiotic state in seeds and to multiply and reproduce again; and the limited effectiveness of the most commonly used chemical control agents, such as copper-based pesticides, due to the emergence and spread of resistant strains [1,2,4,5,6,7,8,9]. 

Furthermore, according to many researchers, as reviewed by La Torre and coworkers [10], the extensive and widespread use of copper pesticides can be considered harmful due to several reasons. As a heavy metal, copper is undegradable, and therefore it accumulates in soil, especially in the surface layers. Thus, copper-based pesticides can be considered as toxic environmental contaminants. High copper levels in soil can negatively affect soil microorganisms (nitrogen-fixing bacteria, nitrifying bacteria, and denitrifying bacteria), as well as macroorganisms [10]. This places copper compounds in the group of broad-spectrum products, i.e., they affect not only the target phytopathogenic bacteria but also the total microflora in the soil. A number of reports on the decreasing susceptibility of many phytopathogenic bacterial species to copper pesticides, including bacterial spot causing xanthomonads (BSCX), are available [4,11]. Moreover, copper plant protection products have been listed as candidates for substitution, which presumes their gradual replacement with environmentally friendly products [12]. All the above facts are a solid basis for reconsidering the use of copper-based pesticides in agriculture, and they challenge scientists to develop alternative plant protection products such as bacteriophage-based biopreparations. The advantages of phage preparations over conventional chemical preparations could be summarized as follows: they do not pollute the environment; there is no evidence that they affect organisms other than bacteria; and phages are capable of infecting one particular bacterial species (in some cases even at the strain level) or several closely related species, which makes them safe for the accompanying microflora. All these beneficial characteristics of bacteriophages are of great importance for the effective and targeted application of phage preparations either in human medicine or in agriculture [13]. 

Deep and extensive studies on the nature of bacteriophages and their role in combating severe bacterial plant diseases have been carried out in the last 20 years or so. Hopeful results regarding phage biocontrol have been reported for several devastating bacterial plant diseases, such as: potato soft rot [14], bacterial wilt on tomato [15], Pierce’s disease on grapevines [16], leaf blight in onion [17], soft rot on lettuce [18], bacterial blight on leek [19], and fire blight on pears and apples [20]. Nakayinga et al. summarized the data concerning the diversity of phages infecting different *Xanthomonas* species [21]. There have only been a few reports on the isolation, characterization, and biocontrol potential evaluation of phages effective against bacterial-spot-causing xanthomonads: phages infecting *Xanthomonas campestris* pv. *vesicatoria* [22,23,24]; phages infecting *X. euvesicatoria* (*Myoviridae* phages isolated in Serbia) [25,26]; phages infecting *X. vesicatoria* (an *Inoviridae* phage [27], a *Myoviridae* phage [28], and one presumptive *Podoviridae* phage) [29]; phages infecting *X. perforans* (unidentified phages) [30]; and phages infecting *X. gardneri* (the only report is for one broad-host-range (polyvalent) phage) [29]. Moreover, according to GenBank database, there is too scarce data on the genome organization of *Xanthomonas* phages, with only 134 fully sequenced genomes compared to 133 for *Escherichia coli* phages, 543 for *Acinetobacter* phages, 231 for *Erwinia* phages, etc. (NCBI). Out of these 134 fully sequenced genomes, only five are from *Xanthomonas* phages associated with bacterial spot xanthomonads: one *X. euvesicatoria* phage (access. number NC_054460); one *X. campestris* pv. *vesicatoria* phage (access. number MH206183); and three *X. vesicatoria* phages (access. numbers NC_017981, MN335248, and NC_054461).

It is known that the rhizosphere and leaf surfaces are the main areas for the application of phage-based biopesticides. Studies reporting phage survival after application in soil and the phyllosphere have been reported for phages infecting *Ralstonia solanacearum*, *Xanthomonas oryzae* pv. *oryzae*, *X. campestris* pv. *vesicatoria*, *X. euvesicatoria*, and *X. perforans* [22,26,30,31,32,33,34]. However, many factors can impact phage persistence and efficacy in these environments: UV radiation, temperature, pH, humidity, the type of soil structure, etc. [13,23,35]. Thus, whether applied in the rhizosphere or phyllosphere, phages’ tolerance to fluctuations in different environmental factors must be screened prior to application. For example, the tolerance of the *X. euvesicatoria* phage KΦ1 to different pH values, storage temperatures, and UV radiation in vitro has been reported [26]. The same characteristics have been studied for broad-host-range bacteriophages effective against *X. euvesicatoria*, *X. vesicatoria*, and *X. gardneri* [29]. Perhaps among the less studied characteristics of phages are their survival in the soil after application and their impact on the rhizosphere microbiome of the plant, excluding a few reports [34].

The aim of this paper was the isolation of *X. euvesicatoria*-specific bacteriophages and the determination of several main and specific characteristics concerning their biology and biocontrol potential. 

## 2. Results

### 2.1. Bacteriophage Isolation and Host Range Determination

Eleven bacteriophages were isolated from the soil sample, a description of which is presented in Section 4.2. Phage isolates were randomly isolated from the resulting clear plates on semi-solid agar plates after the cultivation of the soil suspension along with the specific phytopathogenic bacteria. They were purified and named as follows: BsXeu105t/1, BsXeu105t/2, BsXeu105t/3, BsXeu105t/4, BsXeu105t/5, BsXeu269p/1, BsXeu269p/2, BsXeu269p/3, BsXeu269p/4, BsXeu269p/5, and BsXeu269p/6. The host range of 4 of the isolates (BsXeu105t/1, BsXeu105t/2, BsXeu105t/4, and BsXeu269p/3) was determined using 50 phytopathogenic bacterial strains belonging to 4 species causing bacterial spot on tomato and pepper, as well as Pseudomonas syringae pv. tomato. The results showed that the tested phage isolates were capable of infecting all 33 screened *X. euvesicatoria* strains, including the type strain, regardless of their pathotype, race, and source of isolation (host and region). The strains of the remaining four species of phytopathogenic bacteria (X. vesicatoria, X. gardneri, X. perforans, and P. syringaea pv. tomato) were resistant to the studied phages (Table 1).

### 2.2. Characterization of Newly Isolated Bacteriophages

#### 2.2.1. Determination of Plaque and Virion Morphology

**Figure 1:**
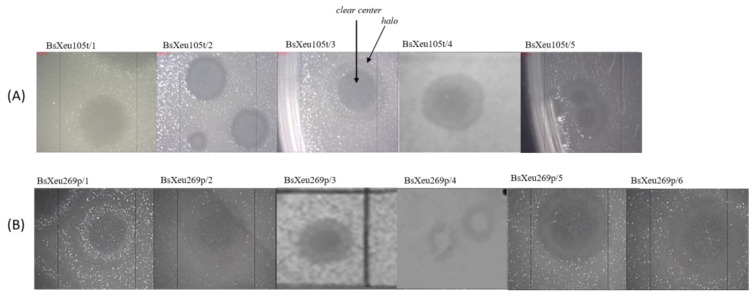
Plaque morphology of the newly isolated bacteriophages. (**A**) Phage isolates BsXeu105t (1 to 5)—host *X. euvesicatoria* strain 105t; (**B**) phage isolates BsXeu269p (1 to 6)—host *X. euvesicatoria* strain 269p.

All newly isolated phages formed plaques with clear centers and a semi- transparent zone (halos) around the center. Slight differences were observed only in plaque dimensions. The diameter of the clear center ranged from 1 to 3.5 mm, with an average value of 2.3 mm, and the diameter of the whole plaque (including the halo) ranged from 3 to 5 mm, with an average value of 4 mm (Figure 1). 

**Figure 2:**
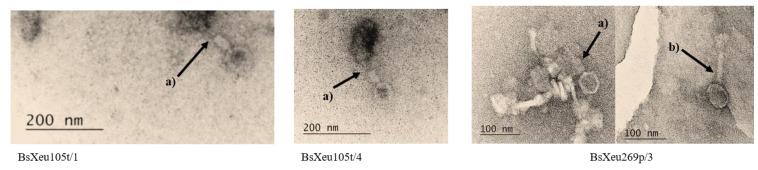
TEM micrographs of the three phage isolates. The arrows indicate: (a) contracted tails and (b) uncontracted tails. Bars: 100 and 200 nm.

The transmission electron microscopy of the three selected phages showed that the virions had icosahedral capsids (approx. 50 nm) and long contractile tails (approx. 100 nm) (Figure 2). These results gave us reason to classify the three isolates into the Caudovirales order (tailed phages) and *Myoviridae* family (phages with long contractile tails), based on the classification of Ackermann [36] and Bradley [37].

#### 2.2.2. Genome Organization of Phage Isolate BsXeu269p/3

**Figure 3:**
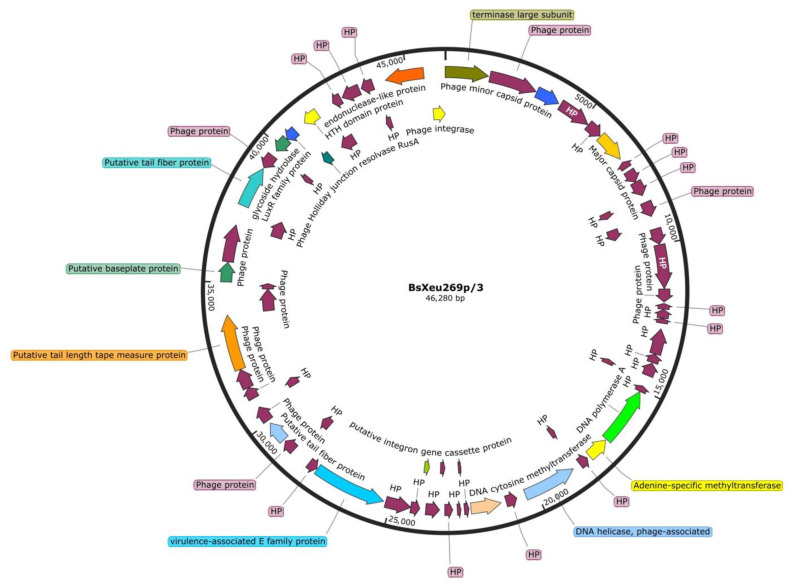
Graphic representation of the circularly permuted genome of phage isolate BsXeu269p/3. The image was generated using “Snap Gene software (www.snapgene.com)”, accessed on 12 June 2022. The colored arrows indicate annotated coding sequences, CDS (28%); HP refers to hypothetical proteins.

The genome organization of phage BsXeu269p/3 was investigated by sequencing. The assembly of the raw sequences, along with the sequences obtained after the PCR amplification of the missing parts between contig 1-2 and 2-1, resulted in the formation of a circular molecule, which we attributed to the circularly permuted nature of the phage genome (Figure 3). The genome of phage BsXeu269p/3 consisted of 46280 bp with 68 open reading frames, 28% identified and annotated proteins, and two 197 bp-long inverted repeats. The complete genome sequence along with annotations was deposited into GenBank with accession number ON996340. We selected the first codon of the terminase gene as the starting point of the assembled sequence and annotated genome. On the positive strand, the terminase gene was followed by genes for structural proteins, such as the minor and major capsid proteins. Next, on the negative strand, the replication module was identified, consisting of genes for helicase, DNA polymerase, and methyltransferases. The positive strand then continued with genes responsible for the formation of the tail structure, such as tail fiber proteins, baseplate proteins, and a tail length tape measure protein. The negative strand also contained genes for a virulence-associated E-family protein, a glycoside hydrolase, a Holliday junction resolvase, and an endonuclease-like protein. On the positive strand, some orf-s were matched to mobile genetic elements from the ACLAME (a CLAssification of Mobile genetic Elements) database, and one gene was assigned as putative integrase [38]. The overall organization of the BsXeu269p/3 genome was highly similar to other *Xanthomonas* phages such as KΦ1 (KPhi1) (acc. no. KY210139 [26]), MYK3 (acc. no. OK275494), and phiXaf18 (acc. no. NC_054461 [28]). The first two phages are positioned in the Unclassified Naesvirus (taxid: 2788421) within the *Myoviridae* family at NCBI (https://www.ncbi.nlm.nih.gov/labs/virus, accessed on 12 June 2022 [39]).

### 2.3. Determination of Phage Survival at 4 °C and −20 °C

**Figure 4:**
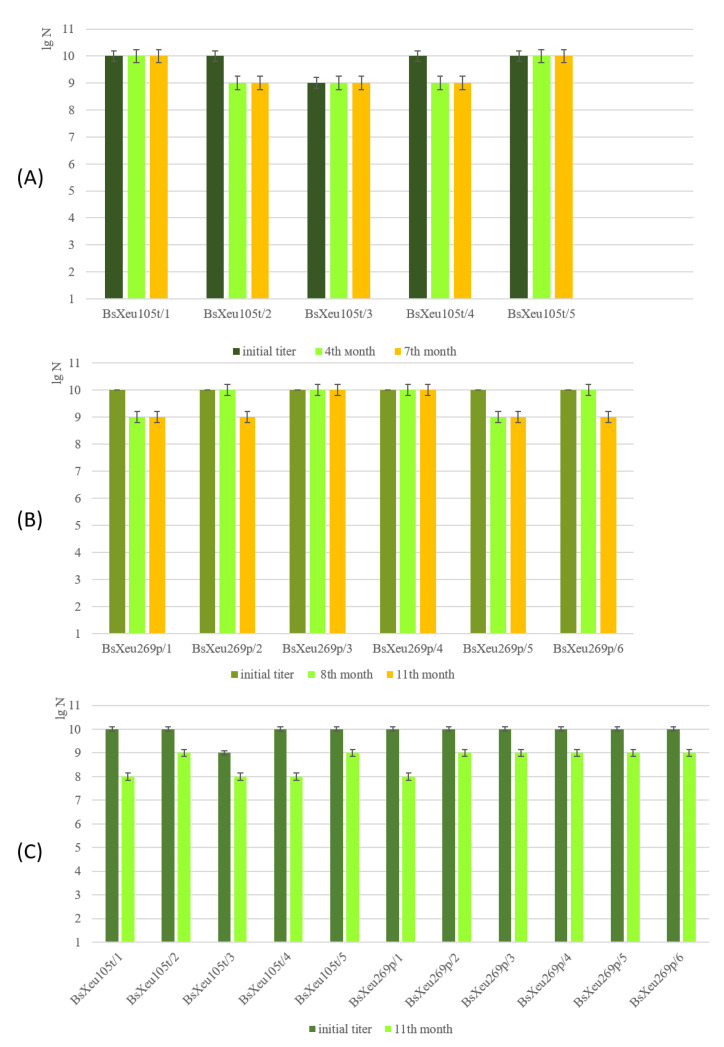
Phage viability at different storage temperatures: (**A**) phage isolates BsXeu105t (1 to 5) stored at 4 °C for 7 months; (**B**) phage isolates BsXeu269p (1 to 6) stored at 4 °C for 11 months; (**C**) storage of all isolates at −20 °C for 11 months.

Pure phage lysates were stored under the chosen temperature conditions for a period of 11 months. The phage titers at 0h were determined and used for subsequent comparison with the titers obtained during the storage period. The concentration of viable phage particles was determined by point testing (at several time intervals for the isolates stored at 4 °C and on the 11th month for those stored at −20°) (Figure 4). The obtained results showed that the isolates differed among themselves in their viability for the testing period. For five of the phage isolates (BsXeu105t/1, BsXeu105t/3, BsXeu105t/5, BsXeu269p/3, and BsXeu269p/4), stored at 4 °C, this temperature did not affect their viability. Their titer at the end of the study was equal to the initial titer (0 h). The titers of the remaining six isolates decreased by a maximum of 1 lg unit. The results obtained after 11 months of storage at −20 °C were roughly similar. Eight of the phage isolates showed a slight decrease in their titer (by 1 lg unit), while in the remaining three isolates (BsXeu105t/1, BsXeu105t/4, and BsXeu269p/1), the decrease in the titer was by 2 lg units (Figure 4).

### 2.4. Determination of Phage Interactions in Soil

#### 2.4.1. Influence of pH on Phage Viability

**Figure 5:**
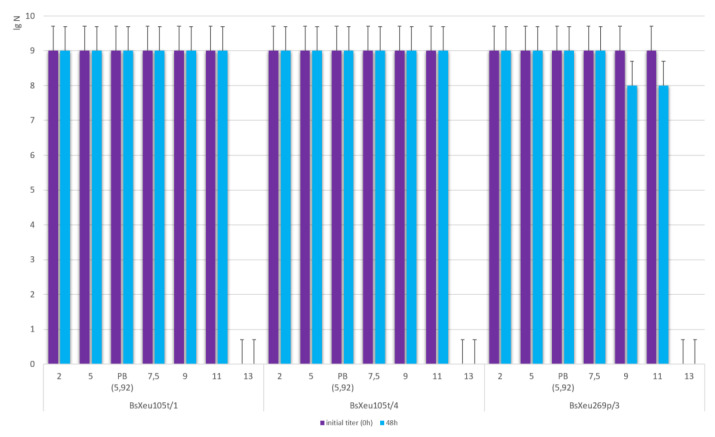
Influence of pH on phage viability; PB—phage buffer.

The selected phage isolates retained 100% of their viability and ability to infect their respective target bacterial strains after 48 h of incubation in buffer solutions of pH 2.0, 5.0, 5.92 (phage buffer), and 7.5, respectively. Incubation at pH 9.0 and 11.0 caused a slight titer decrease of about 1 lg units only for phage isolate BsXeu269p/3 (Figure 5). A strong phagocytic effect was observed immediately after the addition of the phage isolates in a buffer of pH 13.0, as no viable phage particles were detected after the analysis performed at 0 h. Additionally, the influence of different pH values on the bacterial host strains (*X. euvesicatoria* strains 105t and 269p) was investigated. We found that an inhibitory effect was only observed when applying undiluted buffers with pH 13.0 for both strains, as well as with pH 2.0 for strain 105t (Supplementary Figure S1—phage viability after incubation in buffers with different pH).

#### 2.4.2. Measuring the Phage Survival in Soil Samples

The ability of phage isolate BsXeu269p/3 to retain its lytic activity in soil was studied (described in Section 4.6.2). Viable phage particles were detected only on the 55th day from the beginning of the experiment, and their titer was drastically lower (by 8 lg units) compared to the initial titer (10^10^ pfu/mL). No viable phages were detected on the 62nd day.

#### 2.4.3. Influence of BsXeu269p/3 on Beneficial Rhizosphere Microorganisms 

In order to investigate the influence of the phages on the beneficial rhizosphere microflora, we chose the phage isolate BsXeu269p/3. We studied the influence of the phage on 4 functional groups of microorganisms (aerobic heterotrophs and nitrogen-fixing, ammonifying, and denitrifying bacteria) mainly related to the nitrogen cycle in the soil. For this purpose, the total number of these groups was previously determined in the soil sample used and is presented in Table 2. Nitrifying bacteria were not detected in the soil sample; therefore, the influence of the phage isolate on this group of microorganisms was not investigated. The obtained results showed that the studied bacteriophage did not inhibit the growth of heterotrophic and nitrogen-fixing bacteria, since no clear plaques were formed after cultivation. The absence of a negative effect on the ammonifying and denitrifying bacteria was also registered. Their counts after adding viable phages to their culture medium were the same as the initial counts previously found in the soil—10^5^ and 10^4^ MPN/mL, respectively (Table 2).

## 3. Discussion

Bacterial spot disease in tomato and pepper plants is spread worldwide especially in countries with warm and humid climates. Disease management is difficult due to increasing bacterial resistance to conventionally used copper-based pesticides [4,11]. Therefore, researchers’ efforts are focused on the development of alternative means for the effective control of bacterioses. Bio-preparations such as bacteriophage-based products are considered a reliable alternative. The present study focused on the isolation, characterization (phenotypic and genotypic), and evaluation of the potential of newly isolated phages as biocontrol agents targeting the causative agent of spot scab on both pepper and tomato in Bulgaria—*X. euvesicatoria*. This study was a continuation of our previous work [29].

In this study, the main objective was to isolate and propagate phages specific to *X. euvesicatoria* (bacterial spot pathogen on tomato and pepper) and then characterize them according to parameters that are relevant for their further study, with a view to future application as biocontrol agents. In this paper, 11 bacteriophages isolated with the bacterial host *X. euvescatoria* are reported. The host range of four of them was determined, and the results showed that they could infect all strains of the species, regardless of the host and the region from which they were isolated. According to the widely accepted concept of host range determination, these phage isolates could be defined as broad-host-range phages [43]. To our knowledge, there are only two reports on the isolation and characterization of such phages effective against *X. euvesicatoria* in Serbia [25,26]. The phages we reported were isolated from the rhizosphere soil of a tomato plant with symptoms of bacterial spot, while the majority of the phages isolated in Serbia were from soils where pepper was grown [25]. So far, *X. euvesicatoria* has been mainly reported as a bacterial spot pathogen on pepper [1], so the rhizosphere soil of these plants could be a good source for the isolation of *X. euvesicatoria*-specific phages (XESP). This difference in the source of XESP isolation could be explained by the fact that in Bulgaria this pathogen infects tomatoes too [7]. In our case, the rhizosphere of the infected tomato plants could also be used as a source for the isolation of XESP. Elsewhere, the rhizosphere soil of healthy tomato plants was reported by our team as a source of isolation for phages infecting *X. vesicatoria* and *X. gardneri* [29].

The morphology of plaques is an essential characteristic. All our phage isolates formed approximately identical plaques, as the differences were observed only in plaque size. The formation of clear plaques is typical for lytic phages [44]. The presence of a halo can be considered evidence for the action of phage-induced soluble enzymes (depolymerases), which degrade the bacterial capsule exopolysaccharides upon phage infection [44]. Similar conclusions were drawn for two *X. euvesicatoria* phages by Gasic et al. [25]. *X. euvesicatoria*, as member of the *Xanthomonas* genus, formed extracellular polysaccharide xanthan [45], and these results were somewhat expected. The induction of polysaccharide depolymerases has been demonstrated for several capsule-forming bacterial species during phage infection: *Erwinia amylovora* phage f-Ea1h [46], *Pseudomonas aeruginosa* [47], *Escherichia coli K12* [48], and *Rhizobium trifolii* [49]. However, *Xanthomonas* phages forming clear plaques without a halo have also been reported [29].

Some publications have claimed that the plaque size and capsid size are inversely related. Phages with a smaller capsid formed a large plaque in [44]. The authors revealed that the majority of Myophages formed plaques smaller in diameter (≤1 mm) due to their bigger heads. On the other hand, in our study, the selected phage isolates that we classified as members of the *Myoviridae* family had relatively small capsids (approx. 50 nm), but the plaques they formed were relatively big (4 mm). 

Although the genome of BsXeu269p/3 was best represented as a circular molecule, this phage has a physically rather linear, although circularly permuted genome, as in other representatives of the *Myoviridae* family, such as T4-like and P1-like viruses [50]. Circular permutation has been previously reported for the genome of the *X. vesicatoria* phage phiXaf18 [28]. The initial shotgun sequencing and assembly resulted in the construction of two contigs (1 and 2). After PCR amplification with primers designed in this study (Table 3), sequences were also obtained for each potential gap for both orders of contigs (1-2 and 2-1), suggesting a circular structure for the sequenced DNA molecule. The comparison of the two gap sequences showed that they were identical and represented two inverted repeats within the genome, also explaining the gaps in the initial assembly process. We found similar inverted repeats by sequence and location in the genomes of the closely related phages KΦ1, MYK3, and phiXaf18. The fact that in the BsXeu269p/3 genome a putative gene for a Holliday junction resolvase was identified, and inverted repeats in the formation of Holliday junctions in DNA [51] were involved, suggests that the inverted repeats found in our phage genome and its closest relatives might be a potential site for DNA recombination. The genome of BsXeu269p/3 contained two putative methyltransferases, adenine- and a cytosine-specific, respectively, suggesting that the phage DNA might be heavily methylated to avoid the restriction–modification system defense in the host. Although the potential presence of an integrase gene within the genome of our phage was identified, the phage was clearly lytic, bringing doubt as to whether the integrase was functional.

Different strategies have been studied for the long-term storage of pure or purified phage lysates (4 °C, −20 °C, −80 °C), but the efficacy of each method varies and depends on the phages themselves [52]. Generally, the storage of pure phage lysates at 4 °C has been considered a reliable practice. It has been reported that tailed phages and filamentous or icosahedral phages remain viable under these conditions for periods over 5–10 years, even over 32 years [52]. Our results fully corresponded with the findings of the authors mentioned above, since the maximum decrease in the phage titer over the period studied herein was 1 lg unit. Similar results were previously reported for other *Xanthomonas* phages [26,29,53]. However, the low efficacy of these storage conditions has been reported for *Xanthomonas arboricola* phages [54]. Deep freezing (−80 °C) as a long-term storage approach has been found to be less reliable than storage at 4 °C. Rapid inactivation during freezing has been demonstrated for several phages [52], but we should note that the phages we studied did not lose their viability when stored at −20 °C for 11 months.

Among the main strategies for the application of phage-based biopreparation in agriculture is administration via the rhizosphere or phyllosphere of plants. The phyllosphere is the part of the plant that is exposed to the action of a number of environmental factors such as UV radiation, wind, rain, and drought. These factors have been reported to affect the survival of phages on the leaf surface, thereby reducing their efficiency [24,35]. In this regard, the application of phage preparations in the plant rhizosphere could be a better strategy to control phytopathogenic bacteria, even if they cause leaf diseases [20,34,55]. Therefore, it is important to determine certain characteristics of the phages that are relevant to their application in the soil, such as their pH tolerance (with respect to their application in soils with different pH values), their persistence in the soil in the absence or presence of the specific host (in respect to their longer-term activity in the soil), and their influence on the beneficial rhizosphere microbiome of the plant (in respect to their safe application). Among the phages isolated herein, some showed tolerance to very low pH values (pH 2.0), and others were tolerant to high pH values (pH 11.0). These characteristics revealed their potential for use in soils with a wide pH range. Such tolerance to very low and high pH values is not widespread among phages. Gasic et al. [26] reported that phage KΦ1 was completely inactivated after 24 h of storage in buffers of pH 2.0 and pH 12.0. According to Nakayinga and coworkers [21], the pH tolerance range of known *Xanthomonas* phages is between 5.0 and 11.0, with a few exceptions whereby phages have shown resistance to lower pH values (pH 4.0). To a large extent, our results were consistent with those reported by the latter authors. However, data demonstrating phage tolerance to pH 2.0 could also be found for other *Myoviridae* phages specific to *P. aeruginosa* и *E. coli* [44].

Another important feature of phages intended for soil application is their long-term persistence. In this study, we selected the phage isolate BsXeu269p/3, which was inoculated into a soil sample under in vitro conditions in the absence of its host, *X. euvesicatoria*. The experiment was thus designed to determine the ability of the phage to remain viable under the conditions described. Furthermore, *X. euvesicatoria* is not a soil-borne phytopathogenic bacterium, so it can only persist in the soil for a limited period of time. Thus, we conducted an experiment aiming to more closely simulate natural soil conditions. The results obtained gave us reason to conclude that this phage would be suitable for soil application, since under these conditions it remained viable for 55 days. Similar results have been previously reported for several phages specific to *E. amylovora*, *R. solanacearum*, *X. perforans*, *X. euvesicatoria*, and *X. oryzae*, which can migrate from roots to the aerial parts of plants when applied to the rhizosphere [20,32,34,55].

Rhizosphere microorganisms play a key role in plant growth. Therefore, it is important that their structure and function are not negatively affected during the application of plant disease control practices in agriculture. Copper-based preparations kill not only the target phytopathogenic bacteria, but also many microorganisms that are beneficial to the plant. Bacteriophages are considered safe in this regard due to their high specificity towards the target microorganism. However, before a bacteriophage is proposed as a biocontrol agent, its impact on non-target microbiota should be investigated to demonstrate its safety. In our study, we demonstrated the safety of one phage isolate (BsXeu269p/3) against four groups of rhizosphere bacteria. 

## 4. Materials and Methods

### 4.1. Phytopathogenic Bacteria

Fifty phytopathogenic bacterial strains belonging to four species (*X. vesicatoria*, *X. euvesicatoria*, *X. gardneri*, and *P. syringae* pv. *tomato*) were used in this study (Table 1). All used strains were isolated from different regions in Bulgaria and identified and characterized in our previous works [4,5,7,56]. Five types of bacterial cultures were used as controls in the study (Table 1). 

### 4.2. Soil Sample and Bacteriophage Isolation

The soil sample was collected in 2018 from the rhizosphere of tomato plants with symptoms of bacterial spot. Sampling was carried out in a private vegetable garden in the village of Novo Panicharevo, Burgas region, southeastern Bulgaria. Two *X. euvesicatoria* strains (strains 105t and 269p isolated from infected tomato and pepper plants, respectively) were used as host microorganisms for bacteriophage isolation (Table 1). The bacteriophages were isolated by a double agar overlay plaque assay (DAOPA), according to a procedure described before [29].

### 4.3. Phage Purification and Host Range Determination

Pure bacteriophage cultures were obtained in LB broth with their target bacterium, according to the procedure described previously by Kizheva et al. [29], and stored at 4 °C as pure phage lysates. The determination of the host range of the four newly isolated bacteriophages was carried out by a spot-test assay [57]. Four phytopathogenic bacterial species (*X. euvesicatoria* (32 strains), *X. vesicatoria* (14 strains), *X. gardneri* (3 strains), and *P. syringae* pv. *tomato* (1 strain)) and five types of cultures, shown in Table 1, were used as host bacteria. The susceptibility of the host strains to the phage isolates was determined based on the appearance of clear plaques from bacterial lysis. The assay was performed in triplicate.

### 4.4. Characterization of Bacteriophages

#### 4.4.1. Determination of Phage Plaque Morphology

The morphology of the plaques produced by the phages on the surface of the semi-solid agar medium inoculated with the target bacterium was determined by DAOPA. *X. euvesicatoria* strains 105t and 269p were used as host bacteria for 11 phage isolates, 5 of which were designated as BsXeu105t and 6 as BsXeu269p. The analysis was performed by measuring the diameter of the plaques and the determination of the type of plaque center and edges.

#### 4.4.2. Determination of Virion Morphology

The virion morphology of three phage isolates (BsXeu105t/1, BsXeu105t/4, and BsXeu269p/3) was investigated by transmission electron microscopy (TEM). The bacteriophages were propagated on a semi-solid agar medium (Nutrient Agar (NA), Merck KGaA, Darmstadt, Germany) by DAOPA along with their target bacterium. Fresh phage suspensions were prepared in phage buffer [58], according to the procedure described in one of our previous works [29]. Fifty microliters of each resulting phage suspension (10^9^ pfu/mL) was used for preparing the negatively stained formvar-coated grids for observation by TEM [59]. The observations were carried out using a transmission electron microscope JEOL JEM 2100 operating at 200 kV (JEOL Ltd., Tokyo, Japan). 

#### 4.4.3. Sequencing and Bioinformatic Analyses of the Genome of Phage BsXeu269p/3

Fresh phage suspension was obtained after the propagation of the phage isolate BsXeu269p/3 with its specific host on NA medium by DAOPA. Five milliliters of phage buffer [58] was poured onto the surface of the resulting phage culture in a petri dish, which was then incubated on a rotary shaker at room temperature for 1.5 h. The resulting suspension was filtered through a membrane filter (Minisart^®^ Syringe Filter 28 mm, pore size 0.22 μm, Sartorius, 37079 Goettingen, Germany) to completely remove residual bacterial cells; then, it was used for the extraction of phage genetic material by a NORGEN Phage DNA isolation Kit (Norgen Biotec Corp., Thorold, Canada). The sequences were obtained after whole-genome sequencing by Illumina NovaSeq PE150 in Novogene (Novogene (UK) Company Limited, Cambridge, CB4 0FW, United Kingdom).

Raw sequences were assembled into contigs (contig 1 and 2) using Unicycler (Galaxy Version 0.4.8.0) [60]. Two primer pairs, described in Table 3, were designed for the amplification and subsequent sequencing of the gaps between Contigs 1-2 and 2-1. The PCR amplifications were carried out in TECHNE TC-312 apparatus under the following conditions: initial DNA denaturation at 95 °C for 10 min followed by 30 cycles of denaturation at 94 °C for 45 s; annealing at 56 °C and 58° for 45 s (for YK2F2/YK2R2 and YK2F3/YK2R3, respectively); elongation at 72 °C for 45 s; and final elongation at 72 °C for 5 min. The PCR products were separated by 1.5% agarose gel electrophoresis and stained with ethidium bromide. The final assembly of the genome was carried out with CLC Sequence Viewer 6.6.2 (www.clcbio.com, accessed on 12 May 2022). The genome annotation was performed manually and using the online platform RAST [61].

### 4.5. Determination of Phage Survival at Different Storage Temperatures

The determination of phage survival at different storage temperatures (4 °C and −20 °C) was carried out using pure phage lysates, obtained after propagation in LB medium. Glycerol solution (30% final concentration) was used as a cryoprotectant for phage lysates stored at −20 °C. Phage titers were determined by a spot-testing assay, and the determination was performed at various time intervals for the different phage isolates. The experiments were carried out in triplicate.

### 4.6. Determination of Phage Interactions in Soil

#### 4.6.1. Determination of the Influence of pH on Phage Viability

Basic buffer solution with pH 5.92 was prepared as described by Msimbira et al. [58]. It was used for the preparation of six buffer solutions, with pH values of 2.0, 5.0, 7.5, 9.0, 11.0, and 13.0, respectively. Three phage isolates (BsXeu105t/1, BsXeu105t/4, and BsXeu269p/3) were propagated by DAOPA at 28 °C for 24 h. *X. euvesicatoria* strains 105t and 269p were used as host strains. In order to obtain a pure phage suspension, 10 mL of each buffer solution was poured on the surface of the obtained phage cultures in petri dishes and incubated on a rotary shaker at room temperature for 1.5 h. At this step, only viable phage particles passed from the medium surface into the buffer solutions. The resulting phage suspensions were filtered through a membrane filter (pore size 0.22 μm) to completely remove the residual bacterial cells. The effect of the pH on phage viability was evaluated based on the difference in phage titer at 0 h, and that at 48 h was determined by a spot-testing assay, as follows: a series of tenfold dilutions of the phage suspensions were prepared, and then 10 µL of each dilution was spotted on the surface of the semi-solid agar (NA) medium along with bacterial hosts, followed by cultivation at 28 °C for 24 h. The sensitivity of the host bacteria to different pH values (controls) was determined using the same buffer solutions but in the absence of phage particles. A series of tenfold dilutions of the buffer solutions were prepared, and 10 µL of each dilution was spotted on the solid agar medium surface inoculated with the host bacterium (100 µL–10^8^ cfu/mL). All experiments were performed in duplicate. The design of the analysis is illustrated in Supplementary Figure S2—Design of the experiment investigating the effect of pH on the phage viability. 

#### 4.6.2. Measuring the Phage Survival in Soil Samples without Specific Host

The soil sample was collected from the rhizosphere of a healthy tomato plant in 2018 from a private vegetable garden in the village of Novo Panicharevo, Primorsko municipality, Burgas region, southeastern Bulgaria. The sample was stored in a sterile plastic jar at 4 °C and immediately transported to the laboratory, where it was analyzed. 

To determine the time limit of phage survival in soil, we used a soil sample free of naturally occurring *X. euvesicatoria*-specific phages. Pure phage lysate was obtained for the selected phage isolate (BsXeu269p/3) in LB medium. The initial titer (10^10^ pfu/mL) of the resulting phage culture was determined by a spot-test assay. Ten milliliters of the phage lysate was added to a 30 g soil sample (in triplicate) in a sterile plastic jar and incubated at room temperature for a period of 62 days. The persistence of the viable phage particles was studied by DAOPA (host—*X. euvesicatoria* strain 269p), and the phage titers on the 55th and 62nd day of incubation were compared to the initial phage titer.

#### 4.6.3. Influence of Selected Phage Isolate on Beneficial Rhizosphere Microorganisms

The total microbial count (control) of beneficial rhizosphere bacteria (heterotrophs and nitrogen-fixing, ammonifying, nitrifying, and denitrifying bacteria) was calculated in a native soil suspension prepared in saline (10 g soil/90 mL saline). The media, methods, and cultivation conditions applied in the analyses are listed in Table 2. Aerobic heterotrophic bacteria and nitrogen-fixing bacteria were enumerated by the plate counting method. A series of tenfold dilutions (up to 10^−7^) of the soil suspension in saline were prepared, and an aliquot of 100 µL of each dilution (in triplicate) was plated on the surface of the appropriate culture media (Table 2). The number of ammonifying, nitrifying, and denitrifying bacteria was determined by the most probable number method (MPN) [40,41,42].

To determine the influence of the phage isolate BsXeu269p/3 on the beneficial rhizosphere microorganisms, a pure lysate with a titer of 10^10^ pfu/mL was used. The analysis was carried out by two methods—DAOPA and MPN. We used DAOPA to determine the ability of the phage isolate to form clear plaques on a lawn of previously enriched soil heterotrophs (in NAII medium) and nitrogen-fixing bacteria (in nitrogen-free broth medium) (Table 2). A mixture of 100 µL enriched bacterial suspension, 10 mM CaCl_2_, and 3 mL soft NAII (0.45% agar) or NFM agar (0.45% agar) for heterotrophs and nitrogen-fixing bacteria, respectively, was poured onto the surface of the respective agar media in previously prepared petri dishes. The phage lysate was diluted up to 10^−7^, and an aliquot of 10 µL of each dilution was spotted on the surface of the prepared double agar plates, which were cultivated at 28 °C for 24 h. The formation of clear plaques after cultivation indicated the presence of heterotrophic and nitrogen-fixing bacteria susceptible to the phage isolate. The MPN method was applied to study the ability of the phage isolate to reduce (by lysis) the amount of soil microorganisms in liquid media (Table 2). A mixture of 100 µL soil suspension (diluted to 10^−7^), 100 µL pure phage lysate (10^10^ pfu/mL), and 10 mM CaCl_2_ was added to each test tube containing liquid media. 

## 5. Conclusions

Our study emphasized the necessity of studying bacteriophages as promising candidates for combating bacterial infections. We believe that our results for the genome organization of phage BsXeu269p/3 and the virion morphology of two more phages will contribute to the study of phage biology in general. Moreover, for the first time in this paper, we provided evidence of the circular permutation of a linear genome among the known *X. euvesicatoria* phages. This, along with the other characteristics possessed by this phage (the ability to destroy cells of the phytopathogenic bacteria *X. euvesicatoria*, long-term viability in nonspecific storage temperatures and in soil samples, wide pH range tolerance, and a lack of lytic activity towards beneficial soil microorganisms), makes it a potential candidate as a biocontrol agent against bacterial spot disease in tomato and pepper.

## Figures and Tables

**Table 1:** Host range of four phage isolates and basic characteristics of the tested phytopathogenic bacteria.

Bacterial Strains						Lytic Activity of Phage Isolates on Tested Bacterial Strains			
№	Strain №	Year of Isolation	Pathotype-Race	Host	Region of Isolation	BsXeu269p/3 AN ^d^ ON996340	BsXeu105t/1	BsXeu105t/2	BsXeu105t/4
***Xanthomonas vesicatoria***									
	NBIMCC ^a^ 2427	NA	ND	NA	NA	−	−	−	−
**1.**	29t	1995	P4T3	tomato	Petrich	−	*−*	−	−
**2.**	31t	1997	T1	tomato	IG, Sofia ^b^	−	−	−	−
**3.**	1b	1999	P6T1	pepper	Lovech	−	−	−	−
**4.**	32t	1999	P4T3	tomato	IG, Sofia ^b^	−	−	−	−
**5.**	43t	2006	P4T2	tomato	Kostinbrod	−	−	−	−
**6.**	60t	2007	T2	tomato	IG, Sofia ^b^	−	−	−	−
**7.**	68t	2010	T3	tomato	MVCRI, Plovdiv ^c^	−	−	−	−
**8.**	73t	2012	T1	tomato	Topolovgrad	−	−	−	−
**9.**	97t	2015	P4T2	tomato	MVCRI, Plovdiv ^c^	−	−	−	−
**10.**	124t	2016	T3	tomato	MVCRI, Plovdiv ^c^	−	−	−	−
**11.**	130t	2016	T2	tomato	Blagoevgrad	−	−	−	−
**12.**	131t	2016	T2	tomato	Blagoevgrad	−	−	−	−
**13.**	132t	2016	P6T1	tomato	Tyulenovo	−	−	−	−
**14.**	41f	2017	T1	tomato	MVCRI, Plovdiv ^c^	−	−	−	−
***Xanthomonas perforans***									
	NBIMCC ^a^ 8729	NA	ND	NA	NA	−	−	−	−
***Xanthomonas euvesicatoria***									
	NBIMCC ^a^ 8731	NA	ND	NA	NA	+	+	+	+
**15.**	67b	2012	P6	pepper	Pavlikeni	+	+	+	+
**16.**	81b	2012	P6	pepper	Shabla	+	+	+	+
**17.**	86b	2012	P6	pepper	Kavarna	+	+	+	+
**18.**	99b	2013	P4T3	pepper	Byala cherkva	+	+	+	+
**19.**	106b	2013	P6T1	pepper	Shabla	+	+	+	+
**20.**	110b	2013	P4T2	pepper	Kavarna	+	+	+	+
**21.**	111b	2013	P0T2	pepper	Kavarna	+	+	+	+
**22.**	105t	2015	P4T1	tomato	Haskovo	+	+	+	+
**23.**	108t	2015	P1T2	tomato	Svilengrad	+	+	+	+
**24.**	269p	2015	P4	pepper	Durankulak	+	+	+	+
**25.**	274p	2015	P0	pepper	Shabla	+	+	+	+
**26.**	116t	2016	T1	tomato	Kostinbrod	+	+	+	+
**27.**	117t	2016	T2	tomato	MVCRI, Plovdiv ^c^	+	+	+	+
**28.**	119t	2016	P4T2	tomato	MVCRI, Plovdiv ^c^	+	+	+	+
**29.**	120t	2016	P0T2	tomato	Sadovo	+	+	+	+
**30.**	136t	2016	P0T2	tomato	Pazardzhik	+	+	+	+
**31.**	137t	2016	P0T3	tomato	Pavlikeni	+	+	+	+
**32.**	306p	2016	P5T2	pepper	Kostinbrod	+	+	+	+
**33.**	308p	2016	P1T3	pepper	Kavarna	+	+	+	+
**34.**	314p	2016	P3T2	pepper	Pavlikeni	+	+	+	+
**35.**	315p	2016	P1T3	pepper	Pavlikeni	+	+	+	+
**36.**	317p	2016	P6	pepper	Durankulak	+	+	+	+
**37.**	318p	2016	P1	pepper	Shabla	+	+	+	+
**38.**	320p	2016	P1	pepper	Tyulenovo	+	+	+	+
**39.**	321p	2016	P1T1	pepper	Kavarna	+	+	+	+
**40.**	326p	2016	P6	pepper	Shabla	+	+	+	+
**41.**	40f	2017	P4T2	tomato	MVCRI, Plovdiv ^c^	+	+	+	+
**42.**	2p_3_	2020	ND	pepper	Kyustendil	+	+	+	+
**43.**	3t_3_	2020	ND	tomato	Kyustendil	+	+	+	+
**44.**	3p_4_	2020	ND	pepper	Kyustendil	+	+	+	+
**45.**	7p_2_	2020	ND	pepper	Kyustendil	+	+	+	+
**46.**	8p_4_	2020	ND	pepper	Kyustendil	+	+	+	+
***Xanthomonas gardneri***									
	NBIMCC ^a^ 8730	NA	ND	NA	NA	−	−	−	−
**47.**	62t	2009	P0T1	tomato	IG, Sofia ^b^	−	−	−	−
**48.**	64t	2009	P0T3	tomato	IG, Sofia ^b^	−	−	−	−
**49.**	77t	2012	T3	tomato	Topolovgrad	−	−	−	−
***Pseudomonas syringae* pv. *tomato***									
**50.**	32f	2017	R0	tomato	Kostinbrod	−	−	−	−
	NBIMCC ^a^ 3374	NA	ND	NA	NA	−	−	−	−

“+”—sensitive strain, plaque formation; “−”—resistant strain, no plaque formation; NA—not applicable; ND—not determined; ^a^ NBIMCC—National Bank for Industrial Microorganisms and Cell Cultures; ^b^ Institute of Genetics (part of the Institute of Plant Physiology and Genetics, since 2010); ^c^ MVCRI—Maritsa Vegetable Crops Research Institute, Plovdiv, Bulgaria; AN ^d^—accession number in GenBank.

**Table 2:** Influence of phage isolate BsXeu269p/3 on rhizosphere bacteria.

Groups of Microorganisms	Media	Enumeration Method	Duration of Cultivation	Bacterial Quantity		Activity of BsXeu269p/3
				In the Native Soil Sample	In the Presence of Phage Isolate	
Heterotrophs	Nutrient agar II (NAII) [40]	Aerobic plate count	28 °C/24 h	10^5^ CFU/mL	ND ^a^	-
Nitrogen-fixing microorganisms	Nitrogen free media agar (NFM) [40]	Aerobic plate count	28 °C/48 h	10^5^ CFU/mL	ND	-
Ammonifying bacteria	Nutrient broth II (NB) + phenol red (0.2% water solution) [40]	Most probable number	28 °C/up to 1 week	10^5^ MPN/mL	10^5^ MNP/mL	-
Nitrifying bacteria	Mineral media broth + phenol red [41]	Most probable number	28 °C/up to 4 weeks	ND	ND	ND
Denitrifying bacteria	Giltay’s medium broth + Bromothymol blue [42]	Most probable number	28 °C/up to 1 week	10^4^ MPN/mL	10^4^ MPN/mL	-

ND ^a^—not detected; “-”—lack of lytic activity.

**Table 3:** Primer pairs used for the amplification of the missing sequences in the genome of phage isolate BsXeu269p/3.

Primers	Target Sequences between Contigs	Sequences 5′–3′	Phage Sample Type	Amplicon Lengths	Source
YK2F2	1-2	GAAGTCGATTAAACCCTCCG	5-fold diluted phage lysate	850 bp	This study
YK2R2		AACTGACCCTGCTTTAGTTC			This study
YK2F3	2-1	GGGTTGTGAGTGTGGATAAA	5-fold diluted phage lysate	350 bp	This study
YK2R3		TAGATTCCCCTGATCGTCAA			This study

## Data Availability

Not applicable.

## Supplementary Materials

The following supporting information can be downloaded at: https://www.mdpi.com/article/10.3390/plants12040947/s1, Supplementary Figure S1: Phage viability after incubation in buffers with different pH. (A) Host bacterium is *X. euvesicatoria* strain 105t (tomato isolate); control petri dishes were inoculated only with host bacteria; (B) host bacterium is *X. euvesivcatoria* strain 269p (pepper isolate); control petri dishes were inoculated only with host bacteria, Supplementary Figure S2: Design of the experiment examining the effect of pH on the phage viability.
