# Supplementary figures and images for: Phenotypic and Genotypic Characterization of Newly Isolated Xanthomonas euvesicatoria-Specific Bacteriophages and Evaluation of Their Biocontrol Potential
Source: Plants (Basel). 2023 Feb 19;12(4):947. doi: 10.3390/plants12040947 (PMC9968065; doi:10.3390/plants12040947)

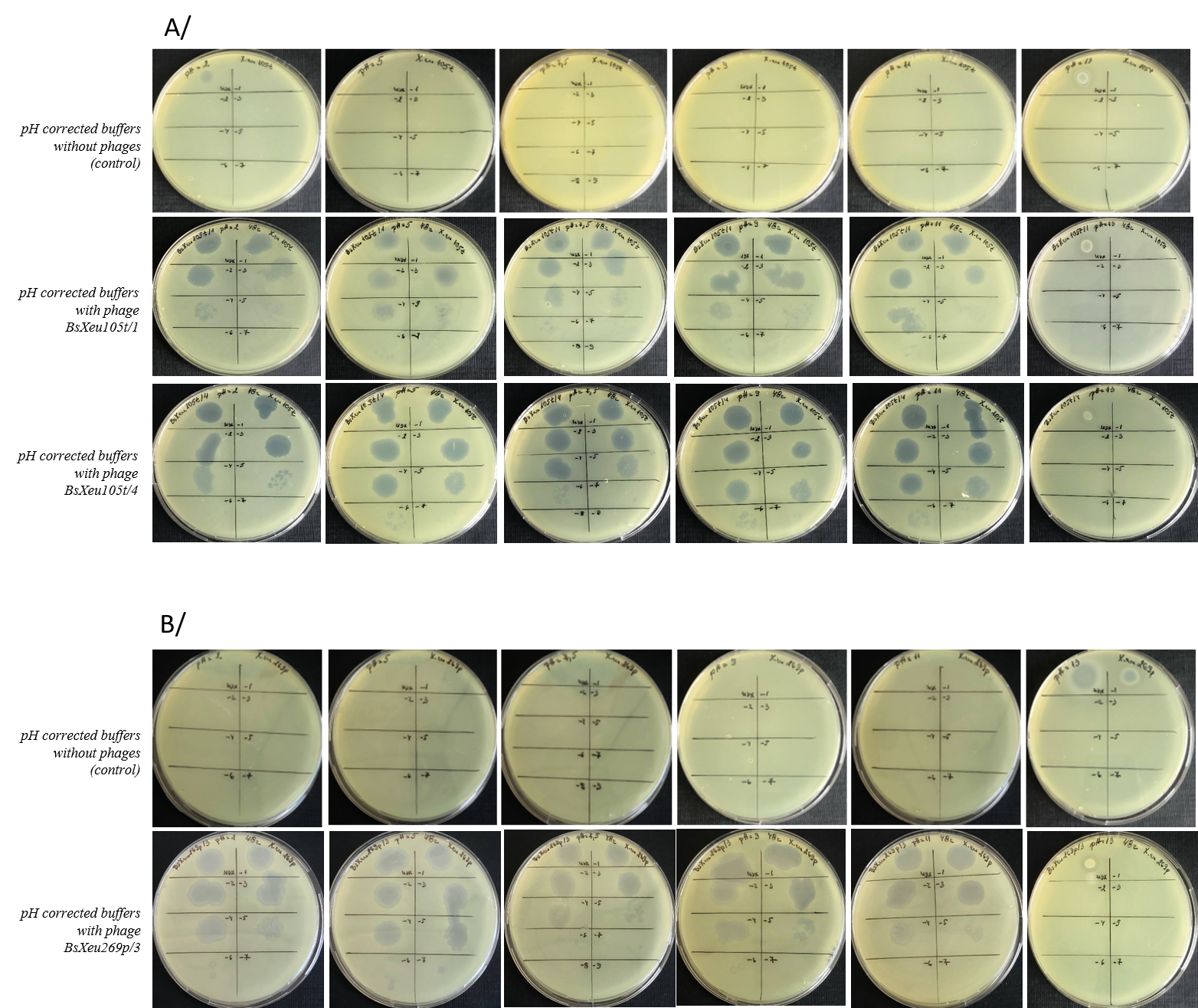

Supplement: Supplementary file 1 [file plants-12-00947-s001.zip › Supplementary figure S1_600 dpi.tiff]

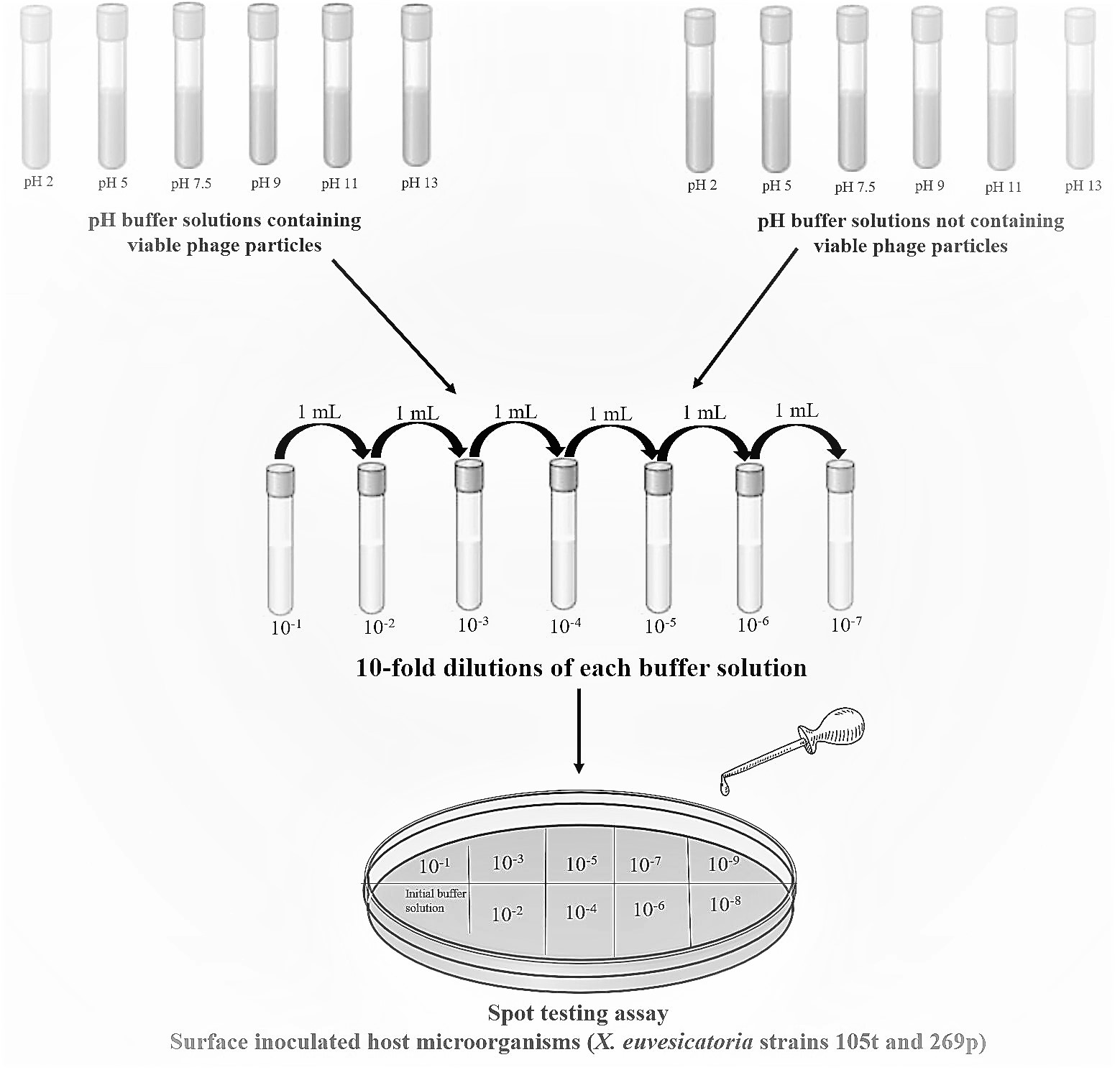

Supplement: Supplementary file 1 [file plants-12-00947-s001.zip › Supplementary firgure S2.tiff]
